# Is there a relationship between psoas impingement and increased trochanteric retroversion?

**DOI:** 10.1093/jhps/hnv024

**Published:** 2015-03-31

**Authors:** Juan Gómez-Hoyos, Ricardo Schröder, Manoj Reddy, Ian J. Palmer, Anthony Khoury, Hal David Martin

**Affiliations:** 1. Hip Preservation Center, Baylor University Medical Center, Dallas, USA; 2. Department of Orthopaedic Surgery, University of Antioquia, Medellin, Colombia, USA; 3. Texas A&M Health Science Center College of Medicine, Dallas, USA; 4. Department of Bioengineering, University of Texas, Arlington, USA

## Abstract

The concept of psoas impingement secondary to a tight or inflamed iliopsoas tendon causing impingement of the anterior labrum during hip extension has been suggested. The purpose of this study was to assess the relationship between the lesser trochanteric version (LTV) in symptomatic patients with psoas impingement as compared with asymptomatic hips. The femoral neck version (FNV) and LTV were evaluated on axial magnetic resonance imaging, as well as the angle between LTV and FNV. Data from 12 symptomatic patients and 250 asymptomatic patients were analysed. The mean, range and standard deviations were calculated. Independent *t*-tests were used to determine differences between groups. The lesser trochanteric retroversion was significantly increased in patients with psoas impingement as compared with asymptomatic hips (−31.1° SD ± 6.5 versus −24.2° ± 11.5, *P* < 0.05). The FNV (9° ± 8.8 versus 14.1° ± 10.7, *P* > 0.05) and the angle between FNV and LTV (40.2° ± 9.7 versus 38.3° ± 9.6, *P* > 0.05) were not significantly different between groups. In conclusion, the lesser trochanteric retroversion is significantly increased in patients with psoas impingement as compared with asymptomatic hips.

## INTRODUCTION

The iliopsoas muscle-tendon unit is formed from a confluence of the psoas and iliacus muscles, which originate from the lumbar vertebrae and pelvis, respectively. The iliopsoas tendon runs beneath the inguinal ligament to insert on to the anteromedial surface of the lesser trochanter [1]. Recently, anatomic variance has been reported that describes a double, and triple-banded iliopsoas tendon in 64.2 and 7.5% of the patients, respectively [2].

Iliopsoas impingement refers to an anterior labral injury due to the direct contact by the iliopsoas tendon. The location of these anterior labral injuries is the 3 o’clock position at the iliopsoas notch without any extension of the injury into the anterosuperior labrum [3–5]. The location of these tears is directly adjacent to the psoas tendon where it lies within the hip joint capsule [6]. These specific acetabular labral injuries have not been attributed to any of the known etiologies of labral injuries (femoroacetabular impingement, trauma, dysplasia, capsular laxity or osteoarthritis) [7].

Psoas impingement may be related to a: (i) tight or inflamed iliopsoas tendon causing impingement of the anterior labrum during hip extension, (ii) psoas scarred or adherent to the anterior capsulo-labral complex, (iii) a hyper-active iliocapsulares muscle causing a traction phenomenon [3].

A tight psoas tendon (a tense psoas tendon lying on the anterior labrum and producing labral contusion) is a common finding during hip arthroscopy in patients with symptomatic psoas impingement, the cause of the tightening has not been yet explored. In a previous study, a higher risk for inferior clinical outcomes was showed in patients with increased FNV, however in those patients, lesser trochanteric version (LTV) was not assessed [8].

The purpose of this study was to assess the amount of LTV in patients with symptomatic psoas impingement and compare that to patients with asymptomatic hips. The hypothesis was that increased retroversion of the lesser trochanter would be found in patients with psoas impingement.

## METHODS

Thirty-four patients with psoas impingement as a main diagnosis during a 7-year period from January 2007 to January 2014 from a single surgeon database were retrospectively reviewed. These cases were compared with 320 magnetic resonance imaging (MRI) of the asymptomatic side in patients who underwent examination for hip pain between January 2006 and January 2010.

The inclusion criteria were, for the symptomatic group: (i) patients with diagnosis of psoas impingement not responsive to conservative treatment, (ii) surgical confirmation of the psoas impingement by injury of the extrarticular margin of the labrum at 3 o’clock and a tight psoas in ∼5°–10° of hip extension with no traction (Supplementary video), (iii) records available for review and confirmation on physical examination and (iv) MRI available for review including McKibbin protocol controlling rotation from the knee to the femoral neck by securing the feet in functional walking position. And the inclusion criteria for the asymptomatic group were: (a) patients who underwent physical examination for unilateral hip pain and (b) MRI with McKibbin protocol available for review of the asymptomatic contralateral side.

The exclusion criteria were: (1) a doubtful diagnosis of psoas impingement on the symptomatic group defined as positive clinical findings of psoas impingement with no evidence of a typical tightness and labral injury at the classic location, (2) prior hip fracture or surgery, (3) lesser trochanteric deformity (previous fracture, bone tumors, etc), (4) low quality or incomplete MRI and (5) incomplete records for review.

The diagnosis of psoas impingement was based on comprehensive history, physical examination and imaging assessment [7]. A surgical finding of a 3 o'clock labral injury beneath to a tight iliopsoas tendon in extension of the hip was considered necessary for detection of psoas impingement.

The medical records of all patients meeting the inclusion and exclusion criteria were reviewed under institutional approval.

Relevant demographic and clinical data, including age, gender, symptomatic side, pain characteristics, clinical tests, duration of symptoms until diagnosis and other associated hip conditions were noted for the symptomatic group. The age, gender and asymptomatic side were noted for the asymptomatic group.

The MRI measurements were obtained through the software Virtual Radiology Enterprise Connect PACS (Philips Healthcare Informatics, Inc.—The Netherlands). The axial cuts of the pelvis and knee obtained from the McKibbin protocol were utilized in this study for the evaluation of the angles. Three parameters were assessed: FNV, LTV and the angle between the femoral neck version and lesser trochanter version (FNVLTVa). The measurements were expressed in degrees.

The angles were measured using a new method based on studies performed by Unlu *et al.* [9] and Shon *et al.* [10] This new method was developed for a previous study about lesser trochanteric anatomy (in press). A previous analysis of the first 30 measurements in asymptomatic hips was made by interclass correlation coefficient (ICCs), with a 95% confidence interval for inter and intra-examiner. The ICCs for all measurements by examiner number one ranged from 0.896 to 0.923. The ICCs for all measurements by examiner number two ranged from 0.788 to 0.952. The ICCs for both examiners together ranged from 0.826 to 0.906.

**Fig. 1.**
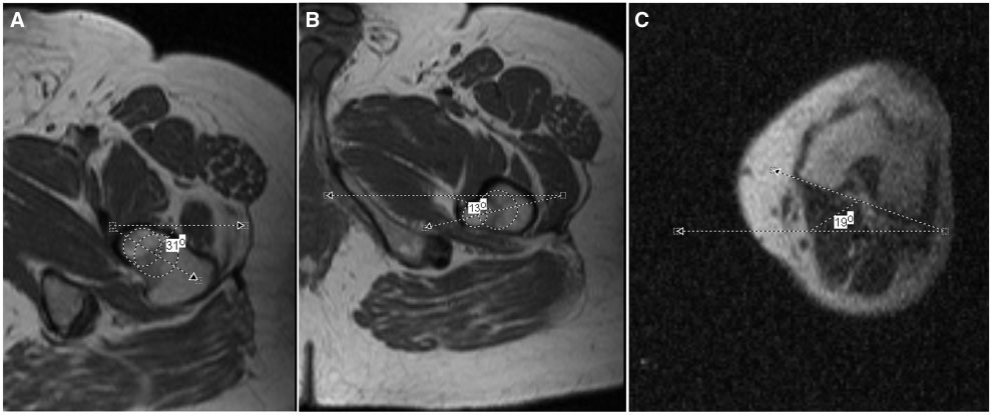
The measurement process of the method was as follows: (i) two centroids were positioned in the body of lesser trochanter or femoral neck, one in the midline of the basis and a second one at the border of the tip as seen in Figs. 1A and B, (ii) the angle of the line passing between the middle of both centroids and horizontal line was called lesser trochanter or femoral neck axis, (iii) the angle between the lesser trochanter axis or femoral neck axis and the posterior condylar axis (Fig. 1C) represented the LTV and FNV, respectively. In this case, FNV = 12 and LTV = −32. A negative value means a retroverted lesser trochanter.

The measurement process of the method was as follows: (i) two centroids were positioned in the body of lesser Trochanter or femoral neck, one in the midline of the basis and a second one at the border of the tip as seen in Figs. 1A and B, (ii) the angle of the line passing between the middle of both centroids and horizontal line was called lesser trochanter or femoral neck axis, (iii) the angle between the lesser trochanter axis or femoral neck axis and the posterior condylar axis (Fig. 1C) represented the LTV and the FNV, respectively.

Additionally, the angle between FNV and LTV was calculated through the formula: FNVLTVa = FNV – LTV.

The mean, range and standard deviations were calculated. Independent *t*-tests were used to determine differences between groups.

## RESULTS

**Fig. 2.**
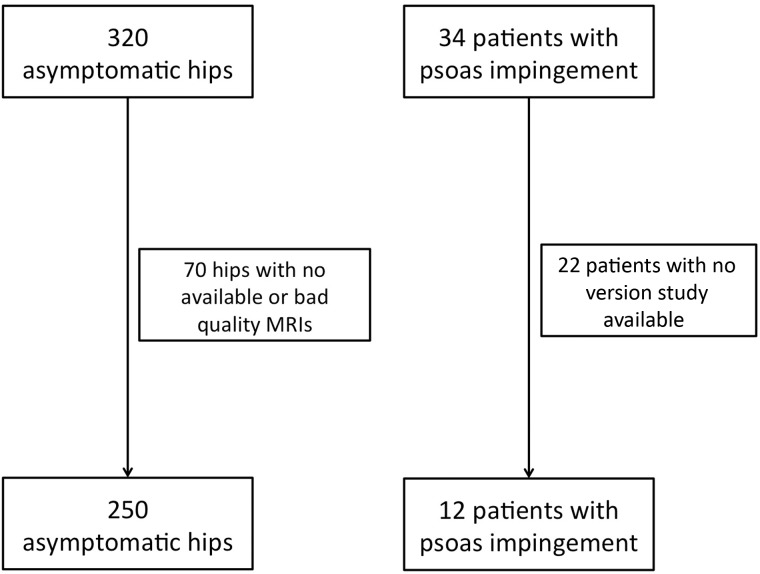
Patient’s selection process.

Twelve patients with psoas impingement and 250 asymptomatic hips met the inclusion criteria (Fig. 2).

The mean age of the included symptomatic patients was 40 years (range, 20 to 57 years) at time of diagnosis. Eight patients were female (75%) and the left side was the most commonly affected (eight cases, 75%). All 12 patients complained of groin pain, difficulty rising from a seated position and limited physical activities. All patients had positive straight leg raise test against resistance and were tender on palpation of the groin. Furthermore, the diagnosis of psoas impingement was confirmed in all cases at arthroscopy by the finding of a 3 o’clock labral injury adjacent to a tight iliopsoas tendon as defined in the introduction section. Other clinical characteristics and associated problems are shown in Table I.

**Table I.** Demographic data and other associated conditions in symptomatic patients

Case	Age (y)	Gender	Side	Other associated conditions
1	32	Female	Left	Femoroacetabular impingement (cam-type)
2	29	Female	Right	None
3	50	Female	Right	Pudendal nerve entrapment
4	20	Male	Right	Femoroacetabular impingement (mixed-type)
5	50	Male	Right	Femoroacetrabular impingement (mixed-type)
6	28	Female	Right	None
7	48	Male	Left	Femoroacetabular impingement (mixed type)
8	26	Male	Right	Femoroacetabular impingement(mixed type)
9	45	Female	Left	None
10	51	Female	Left	None
11	39	Female	Right	Femoral neck retroversion
12	57	Female	Right	Femoroacetabular impingement (mixed-type)

The asymptomatic group included patients with a mean age of 39.48 years (range, 14 to 73 years) at time of diagnosis. One hundred and sixty four patients were female (65.6%) and the right side was the most commonly assessed (140 cases, 56%).

**Fig. 3.**
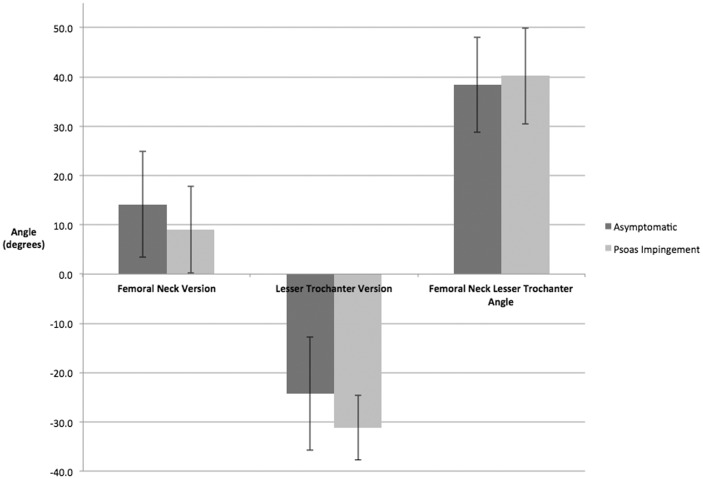
The difference on average and range between groups in FNV and FNVLTVa is similar and non-significant. However, the mean lesser trochanter retroversion is significantly more retroverted in psoas impingement group, with a range of variability more negative and smaller.

The lesser trochanter was significantly (*P* < 0.05) more retroverted in patients with psoas impingement as compared with the asymptomatic group (−31.1° SD ± 6.5 versus −24.2° SD ± 11.5). The FNV between groups and the angle between FNVLTV resulted in no significant differences between the two groups (Table II and Fig. 3). Post-hoc power analysis indicated that the power to detect the described findings was 92.2%.

**Table II.** Comparison between the patients with symptomatic psoas impingement and asymptomatic hips

Group	Symptomatic (*n* = 12)	SD	Asymptomatic (*n* = 250)	SD	Mean difference	*P* value
FNV (°)	9	±8.8	14.1	±10.7	5.1	>0.05
LTV (°)	−31.1	±6.5	−24.2	±11.5	−6.9	<0.05
Angle between the LTV and FNV (°)	40.2	±9.7	38.3	±9.6	1.9	>0.05

SD: standard deviation.

## DISCUSSION

The result of this study suggest that lesser trochanter retroversion is significantly increased in patients with psoas impingement. However, this study found that FNV and the angle between FNALTV are similar to that of the asymptomatic hips.

This study contributes to the understanding of psoas impingement pathomechanics and lends value to the diagnostic approach of patients presenting with groin pain, difficulty rising from seated position and positive straight leg raise test against resistance. Furthermore, the evaluation of the LTV could provide an additional factor to decide the treatment in patients with suspected psoas impingement.

Alpert *et al.* dissected eight hip joints and described the anatomic relationship of the iliopsoas tendon with the acetabular labrum. In all cases, they found the iliopsoas tendon located directly anterior to the anterosuperior capsulolabral complex at the 2 to 3 o’clock position. This study suggests that the close anatomic relationship of the psoas tendon to the anterior capsulolabral complex may lead to labral injury and higher possible incidence when the iliopsoas tendon is tight [11].

From an anatomic point of view, the psoas tendon could be tight due to a large femoral head, an anatomical abnormality around the pectineal eminence (bony prominence beneath the psoas tendon when it passes anterior to the hip), or a more anteverted native or artificial femoral head [12]. Phillipon described the consistent anatomical relationship of the lesser trochanter with the psoas insertion [2]. Considering that the psoas tendon makes an obtuse angle over the pectineal eminence and femoral head, which increases with hip extension, a more retroverted lesser trochanter may elevate contact pressures beneath the tendon thus contributing to the anterior impingement phenomenon.

Although Unlu *et al.* [9] observed a constant relationship between the lesser trochanter and posterior femoral chondyles in 59 hips (34.1° SD ± 3.0°), this study could not confirm similar results. Of the 250 asymptomatic hips assessed in this study with the described method the mean LTV was −24.2° (SD ± 11.5°).

To further show the anatomic variation in LTV, a statistical significant difference was found between asymptomatic hips and psoas impingement hips, with a more negative and smaller range of variability as seen in Fig. 3. Additionally, this finding may indicate that lesser trochanteric retroversion may predispose one to psoas impingement.

Blakenbanker *et al.* recently reported that the MRI is a good tool for detecting psoas impingement when an acetabular labral injury is present at the 3 o’clock position is present [5]. An increased lesser trochanteric retroversion could also contribute to increase the post-test probability of this imaging tool.

To date, most of the studies reporting psoas impingement are related to hip replacements [1, 13–16]. In this case, impingements can be related to malposition or large size of the acetabular component, producing a pulley beneath the tendon [17]. However, in cases of psoas impingement with a good position and a normal size of the acetabular component, a relative retroversion of the lesser trochanter due to an increased anteversion of the femoral component could explain part of the problem.

Iliopsoas impingement may be present in both artificial and natural hips, and as Yoshio *et al.* [12] reported, the most important pulley of the psoas tendon is the prosthetic or native femoral head. Interestingly, the result of the current study found the mean femoral version was similar to the normal value reported in the literature [18].

Furthermore, the angle between FNV and LTV was similar between psoas impingement and asymptomatic hips in this study, suggesting that only the increased retroversion of the lesser trochanter could be an important contributing factor for psoas impingement.

The anatomically retroverted lesser trochanter may help explain why patients with normal anteversion could present with psoas tendon pathology. However, psoas impingement should also be understood as a multifaceted problem that can include scarred or adherent psoas tendon, repetitive traction injury, prosthesis malposition, increased femoral anteversion and hyper-active iliocapsularis muscle [3].

Limitations of this study include: (i) the sample size limits the generalizability of the results, however, psoas impingement is a rare diagnosis even in hip centers and the sample of this report is comparable with previous reports [3], and the Post-Hoc analysis revealed a power of 92.2%, (ii) 22 out of 34 cases had unavailable MRI or non-complete MRI for measuring the angles with the knee as a reference. Exhaustive attempts were made to acquire adequate MRI without success. (iii) Some patients had other associated conditions that could be the cause of the groin pain.

## CONCLUSION

The lesser trochanteric retroversion is significantly increased in patients with symptomatic psoas impingement as compared with asymptomatic hips.

## SUPPLEMENTARY DATA

Supplementary data are available at *Journal of Hip Preservation Surgery* online

## CONFLICT OF INTEREST STATEMENT

None declared.
